# Low Body Mass Index Can Identify Majority of Osteoporotic Inflammatory Bowel Disease Patients Missed by Current Guidelines

**DOI:** 10.1100/2012/807438

**Published:** 2012-05-03

**Authors:** Ashish Atreja, Ashish Aggarwal, Angelo A. Licata, Bret A. Lashner

**Affiliations:** ^1^Department of Gastroenterology, A-31, Digestive Disease Institute, Cleveland Clinic, 9500 Euclid Avenue, Cleveland, OH 44195, USA; ^2^Department of Internal Medicine, Medicine Institute, Cleveland Clinic, Cleveland, OH 44195, USA; ^3^Department of Endocrinology, Center for Space Medicine, Bone Metabolic Institute, Cleveland Clinic, Cleveland, OH 44195, USA

## Abstract

*Background*. Patients with inflammatory bowel disease (IBD) are at high risk of developing osteoporosis. Our objective was to determine the usefulness of IBD guidelines in identifying patients at risk for developing osteoporosis. *Methods*. We utilized institutional repository to identify patients seen in IBD center and extracted data on demographics, disease history, conventional, and nonconventional risk factors for osteoporosis and Dual Energy X-ray Absorptiometry (DXA) findings. *Results*. 59% of patients (1004/1703) in our IBD cohort had at least one risk factor for osteoporosis screening. DXA was documented in 263 patients with indication of screening (provider adherence, 26.2%), and of these, 196 patients had DXA completed (“at-risk” group). Ninety-five patients not meeting guidelines-based risk factors also had DXA completed (“not at-risk” group). 139 (70.9%) patients in “at-risk” group had low BMD, while 51 (53.7%) of “not-at-risk” patients had low BMD. Majority of the patients with osteoporosis (83.3%) missed by the current guidelines had low BMI. Multivariate logistic regression analysis showed that low BMI was the strongest risk factor for osteoporosis (OR 3.07; 95% CI, 1.47–6.42; *P* = 0.003). *Conclusions*. Provider adherence to current guidelines is suboptimal. Low BMI can identify majority of the patients with osteoporosis that are missed by current guidelines.

## 1. Introduction

Inflammatory bowel disease (IBD) is a common disorder, affecting about 1.4 million people in the United States and 2.2 million people in Europe. The incidence of fractures among patients with IBD is reported to be 40% higher than in the general population [1]. Osteoporosis and osteopenia, characterized by low bone mineral density (BMD), are increasingly recognized as common extraintestinal features of IBD that increase fracture risk. Estimates vary based on study populations and location, but in general prevalence of osteopenia and osteoporosis in patients with IBD ranges from 22%–77% and 17%–41% for osteopenia and osteoporosis, respectively [2–4]. The pathogenesis of low BMD in IBD is complex and considered to be multifactorial. Risk factors for the development of low BMD include the general risk factors for osteoporosis such as age, smoking as well as IBD-specific risk factors such as corticosteroid use, malnutrition, small bowel resection, vitamin D (25-hydroxyvitamin D [25-OHD]) deficiency, and proinflammatory cytokines [5, 6]. Recognizing the increased risk for fractures in patients with low BMD, American College of Gastroenterology (ACG) and American Gastroenterology Association (AGA) guidelines recommend screening IBD patients with Dual Energy X-ray Absorptiometry (DXA) if they have one of the following risk factors: postmenopausal state, ongoing corticosteroid treatment, cumulative prior use of corticosteroids exceeding 3 months, history of low-trauma fractures, or age over 60 [7–9]. 

There is limited literature determining the utility of these guidelines in identifying the patients at risk for low BMD. In a study of 100 consecutive patients, Kornbluth et al. showed that among patients who met the AGA criteria for initial DXA screening, osteoporosis was found in 12% and osteopenia in another 44% [10]. While this study showed the positive predictive value of AGA guidelines, it did not specifically assess the negative predictive value of screening criteria proposed by guidelines. In other words, if a patient does not meet the screening guidelines, can he or she still be at risk for low BMD and fractures? This question is especially relevant since these guidelines published many years ago do not take into account clinical risk factors such as low body mass index (BMI) that has been strongly associated with low BMD and increased fracture risk in multiple studies in general population. In fact, the WHO fracture risk predictor model (FRAX) based on data derived from nine cohorts from Europe, North America, Asia, and Australia includes low BMI as an important clinical predictor to predict 10-year probability of fracture [11]. The purpose of this study was to evaluate the utility of osteoporosis screening guidelines in a large outpatient IBD practice, assess provider adherence, and determine the impact of nonconventional risk factors such as low BMI that are not included in current guidelines. 

## 2. Methods

We utilized institutional clinical data repository to identify patients >18 years of age who were seen at least twice in our tertiary care IBD practice and had a diagnosis of Crohn's disease (CD, ICD-9-CM code 555.xx) or ulcerative colitis (UC, ICD-9-CM code 556.xx). We then extracted demographic, laboratory, and DXA information on study patients and reviewed patients' charts for clinical information and data related to conventional (age, steroid use, and postmenopausal status) and nonconventional risk factors (low body mass index, BMI <21 kg/m^2^, total or subtotal colectomy) for low BMD. Any patient with cumulative oral steroid prescriptions lasting greater than 3 months was considered to have “steroid use.” Patients who were found to have unconfirmed UC, CD, or indeterminate colitis in manual review of clinical notes were excluded from the study. World Health Organization (WHO) criteria for low BMD were applied for this analysis [11]. A T score of −1 represents a BMD measurement 1 SD below the mean, and each SD decline in T score is associated with an approximate doubling of relative risk of fracture [12]. T scores between 1 and 2.5 SDs below the average for the reference population were classified as osteopenia. Measurements 2.5 SDs or more below the young adult mean were classified as osteoporosis.

All patients who underwent DXA screening and had BMD measurements available to us were included for further analysis. Differences between the demographics, clinical characteristics, and risk factors for patients with normal and low BMD were determined by Fisher's exact test for categorical variables and Student's *t*-test or Mann-Whitney *U*-test for continuous variables. Variables that appeared to be imbalanced between the two groups were included into the multivariable models. BMD was modeled as T score above or below the cutoff value for osteopenia (i.e., 1 SDs below the young adult mean value). The odds ratio (OR) of low BMD was then estimated in a multivariable logistic regression model. The level of significance was set at 0.05 and analyses were done using SPSS Statistical Software Package (version 16.0, Chicago, IL). The study was approved by Cleveland Clinic Institutional Review Board.

## 3. Results

### 3.1. Demographic and Clinical Baseline Data

**Figure 1:**
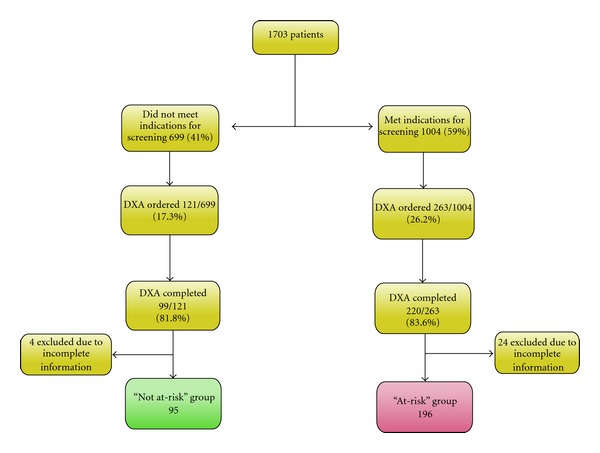
Flow chart of the initial Inflammatory Bowel Disease (IBD) cohort and the final list of patients who did not meet (“not at risk”) and met (“at-risk” group) the indications for osteoporosis screening per the guidelines.

A total of 1703 IBD patients were seen in our IBD center for more than one visit from 2003–2008. Flowchart in Figure 1 shows the categorization of patients in the study. Out of these 1703 patients, 1004 (59%) had at least one indication for DXA scanning as per current guidelines. DXA was ordered or mentioned in electronic health record (EHR) system for 263 out of these 1004 patients (provider adherence 26.2%). Of these 263, 220 (83.6%) patients completed the scan. DXA scan was also ordered in 121 patients who did not have any conventional risk factors (“not at risk” group); of these 99 patients completed the scan. Hence, a total of 319 IBD patients completed at least one DXA scan.

From the 319 patients on who had DXA completed, 28 patients with DXA done outside our health system did not have DXA reports available for confirmation and were excluded from further analysis.  Table 1 shows the demographic characteristics, risk factors, and outcomes of 291 remaining IBD patients, categorized into “at risk” group (those to who met the guidelines, *n* = 196) and “not at risk” group (*n* = 95). 139 (70.9%) of patients in “at-risk” group had low BMD (osteoporosis, 45 (23.0%); osteopenia 94 (47.9%)) while 51 (53.7%) of “not-at-risk” patients had low BMD (osteoporosis, 12 (12.6%), osteopenia 39 (41.1%)). As expected, “not-at-risk” group was more likely to have patients with normal BMD than patients in “at-risk” group (41.1% versus 48%, 0.008). Overall, 42/57 (73.0%) of patients with osteoporosis and 51/133 (38.3%) with osteopenia were documented to be on one of the antiresorptive agents. Bisphosphonates alone or in combination were most commonly used antiresorptive agents in both osteoporosis (41/57, 71.9%) and osteopenia (50/133, 37.6%).

Multivariate logistic regression analysis showed that low BMI was the strongest independent risk factor for osteoporosis (OR 3.07, 95% CI 1.47–6.42) (Table 2). Age (OR 1.02, 95% CI 1.00–1.05) and female gender (OR 2.09; CI 0.99–4.4) were also associated with osteoporosis; however, the relation was not as strong as with low BMI. Disease condition CD or UC was not a predictor of osteoporosis. Subset analysis showed that cigarette use had a variable effect on BMD in CD and UC patients. In CD, there was no association of BMD with cigarette smoking (OR 1.1, 95% CI 0.55–2.19). However, in UC, cigarette smoking was associated with decrease in risk of developing low BMD (OR 0.27, 95% CI 0.1–0.7).

**Figure 2:**
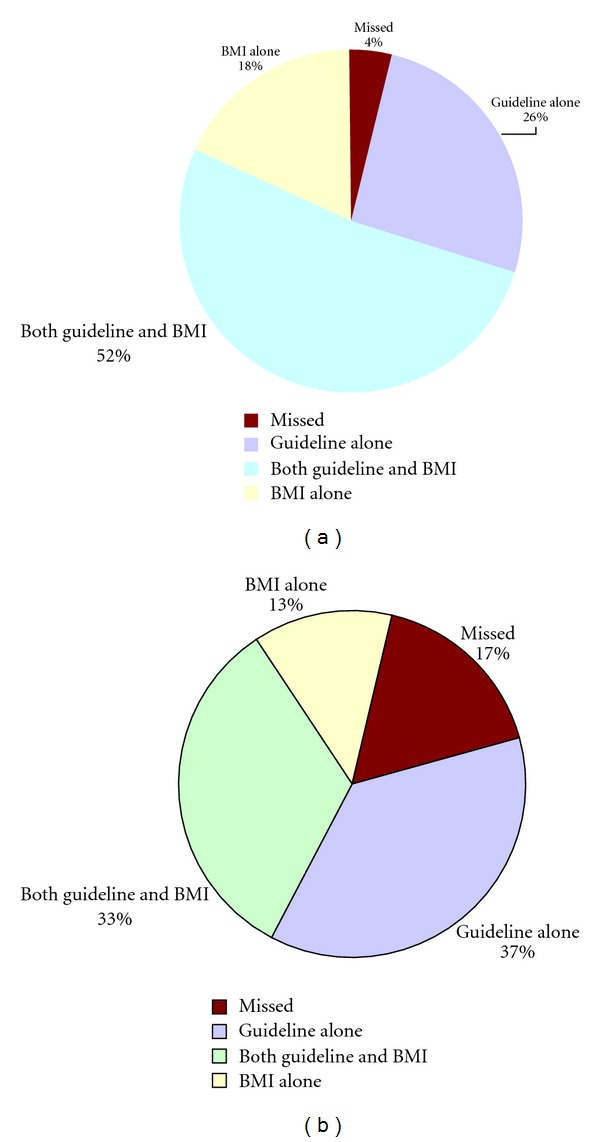
(a) Shows the impact of including low body mass index (BMI) to current guidelines in detecting additional cases of osteoporosis. (b) Shows the impact of including low body mass index (BMI) to current guidelines in detecting additional cases of osteopenia.

Figures 2(a) and 2(b) show a schematic representation of incremental yield of adding low BMI to current guidelines in detecting patients with low BMD in our cohort. As seen in the figures, low BMI alone can yield about the same utility as the current screening guidelines. More specifically, low BMI identified 10 out of 12 (83.3%) osteoporotic patients and 17 out of 39 (43.6%) osteopenic patients missed by the current guidelines. Furthermore, inclusion of low BMI to current guidelines can lead to identification of majority of osteoporosis (53/57, 96.5%) and osteopenic patients (111/133, 83.5%).

## 4. Discussion 

The primary objective of this study was to evaluate the utility of osteoporosis screening guidelines in a large outpatient IBD practice and determine the impact of nonconventional risk factors such as low BMI that are not included in current guidelines. Our study confirmed findings from prior study by Kornbluth et al. about the utility of osteoporosis screening guidelines in IBD population [10]. More than two-thirds of patients (70.9%) in “at risk” group identified by guidelines had low BMD and majority of these patients were started on antiresorptive agents to address low BMD. Age and steroid use were associated with risk of having low BMD. However, about half (53.7%) of patients without the conventional risk factor (“not at-risk” group) had osteopenia or osteoporosis in our study. This shows that negative predictive value of guidelines-based screening approach is not good and a substantial numbers of patients at risk can go undetected if only conventional risk factors are considered. Our multivariate analysis showed that low BMI was the strongest independent risk factor for osteoporosis, with low BMI patients more than three times as likely to have osteoporosis irrespective of the screening guidelines. Addition to low BMI to current guidelines will result in screening of majority of patients found to have low BMD in our cohort.

Our study results are consistent with emerging literature showing low BMI as a strong risk factor for low BMD in both IBD and non-IBD population. In fact, low BMI is included in WHO fracture risk predictor model (FRAX) that is based on data derived from nine international cohorts and validated in may more independent cohorts with a widespread geographic distribution [13]. In 2006, Bartram et al. [14], reported independent association between low BMI and osteoporosis among CD patients in United Kingdom. In a subsequent study in Scottish population, Noble et al. showed a linear correlation between T score at the vertebral spine and BMI in patients with CD [15]. After adjusting for smoking, steroid use, Montreal location, and behavior, BMI remained robust predictor of osteoporosis in CD patients (OR 5.83, CI 1.31–25.94, *P* = 0.021). Our study supports the British Society of Gastroenterology guidelines that low BMI is a strong risk factor for low BMD in not only CD but also in UC patients [16].

Our study also highlights the continued problem of suboptimal physician adherence to osteoporosis screening and management guidelines in IBD patients published in 2003. In 2005, Reddy et al. found that 78% of the IBD patients on chronic steroid therapy did not undergo any BMD scanning or receive any pharmacological therapy for prevention of metabolic bone disease [17]. Wagnon et al. reported that majority of clinicians (57%) did not use the guidelines in management of their IBD patients even in 2009 [18]. Greatest barrier, according to their study, was the perception among clinicians that IBD, and not osteoporosis, should be the focus of the visit. Also, gastroenterologists are not traditionally trained in evaluation and management of metabolic bone disease. Kane and Reddy showed that even after reading the guidelines, only 25% of gastroenterologists felt comfortable managing osteoporosis [19]. Unfortunately, as shown in our study, adherence to guidelines alone will not be sufficient in decreasing fracture risk in our IBD population since the guidelines themselves may miss many patients at risk. Hence, efforts need to be made not only in better adoption of guidelines but also in revising the current guidelines to include nonconventional risk factors such as BMI in assessment of fracture risk. Fortunately, BMI is easy to understand and measure. Majority of electronic health records available today calculate BMI automatically, making it easy to be incorporated into decision making tools such as FRAX. 

Several potential limitations concerning our study findings need to be addressed. First, there may be a selection bias with respect to the patients who underwent DXA screening. Our IBD cohort included a large number of patients who were referred to our tertiary center for management of IBD and its complications. Therefore, they are more likely to have more severe disease and a greater risk for osteoporosis. Furthermore, it is possible that physicians may have chosen to order DXA based on disease severity even if they did not meet criteria for age, steroid use, postmenopausal state, or history of low-trauma fractures. This may have accounted for slightly higher prevalence of low BMD seen in our study as compared to other studies in IBD population [10, 19]. Increased number of referral population may also explain, in part, low number of DXA screenings ordered in our electronic health records. It is possible that physicians discussed DXA screenings and patients underwent DXA at a local facility, outside our health system. We tried to capture outside hospital DXA results by extracting this information from clinical notes documented in our EHR but cannot be sure that all outside hospital DXA results were included in clinical notes. Thirdly, BMD is just one of the many factors that contribute to fracture risk [12, 20, 21]. Studies have shown that a large part of variance in fracture risk is not explained by BMD alone, and low BMI may predict fracture risk independent of BMD too [19, 22]. Hence, fracture risk assessment tools like FRAX that include low BMI as risk factor with or without DXA are increasingly advocated in general population to make treatment decisions in patients at risk for fractures [23]. Future studies looking at applicability of FRAX scores in IBD population and determining the benefits of nutritional intervention in IBD patients with low BMI on bone density and fracture risk will be of great interest. 

In conclusion, this study reveals that current osteoporosis screening guidelines in IBD population can miss a substantial proportion of patients at risk for fractures. Low BMI is an independent and strong risk factor for osteoporosis in both UC and CD patients and should be incorporated in revised guidelines. Future studies should look at applicability of FRAX scores in IBD population and make treatment decisions based on future fracture risk rather than BMD values alone. 

What is current knowledge

- Patients with inflammatory bowel disease (IBD) are at increase risk for osteoporosis and osteopenia.
- Current guidelines recommend screening IBD patients with Dual Energy X-ray Absorptiometry (DXA) if they have one of the following risk factors: postmenopausal state, ongoing corticosteroid treatment, cumulative prior use of corticosteroids exceeding 3 months, history of low trauma fractures, or age over 60.

What is new here

- Guidelines fail to identify many patients who were found to have osteoporosis or osteopenia.
- Low body mass index (BMI) is a strong predictor of low bone mineral density and inclusion of low BMI to current guidelines will help identify majority of IBD patients at risk for osteoporosis or osteopenia.

## Figures and Tables

**Table 1:** Characteristics of patients in the “not at-risk” and “at-risk” groups defined by absence or presence of fulfilling current screening guidelines.

Characteristic	Not at-risk (*n* = 95)	At-risk (*n* = 196)	*P* value
*Demographics*			
Age ± SD (years)	43.6 ± 10.9	48.3 ± 16.2	0.01
Female gender (%)	69.5	58.2	0.15
Ethnicity (%)			0.43
White	90.5	86.2	
African American	5.3	10.3	
Other	4.3	3.5	
*Risk factors (%) *			
Crohn's disease (%)	63.2	63.8	1.00
Smoking (%)			0.17
Current	24.5	14.4	
Past	24.5	39.7	
Colectomy (%)	4.2	8.3	0.26
Partial	30.9	35.3	
Complete	26.6	18.2	
Low body mass index (<21 kg/m^2^) (%)	47.4	48.1	1.00
*Outcome (%)*			0.008
Normal Bone mineral density	46.3	29.1	
Osteopenia	41.1	48.0	0.52
Osteoporosis	12.6	23.0	0.52

Data reported in percentage (%) for categorical variables, mean ± standard deviation (SD) for continuous variables.

**Table 2:** Results of the multivariate logistic regression in our IBD cohort with osteoporosis as the primary outcome.

Variable	Odds ratio	95% CI	Significance (*P*)
Low BMI	3.07	1.47–6.42	0.003
Age	1.02	1.00–1.05	0.05
Female Gender	2.09	0.99–4.4	0.05
Steroid use	1.64	0.79–3.42	0.18
Colectomy	1.7	0.84–3.43	0.14
