# Corrigendum to “Low Body Mass Index Can Identify Majority of Osteoporotic Inflammatory Bowel Disease Patients Missed by Current Guidelines”

**DOI:** 10.1155/2017/3937676

**Published:** 2017-08-14

**Authors:** Ashish Atreja, Ashish Aggarwal, Angelo A. Licata, Bret A. Lashner

**Affiliations:** ^1^Department of Gastroenterology, A-31, Digestive Disease Institute, Cleveland Clinic, 9500 Euclid Avenue, Cleveland, OH 44195, USA; ^2^Department of Internal Medicine, Medicine Institute, Cleveland Clinic, Cleveland, OH 44195, USA; ^3^Department of Endocrinology, Center for Space Medicine, Bone Metabolic Institute, Cleveland Clinic, Cleveland, OH 44195, USA

In the article titled “Low Body Mass Index Can Identify Majority of Osteoporotic Inflammatory Bowel Disease Patients Missed by Current Guidelines” [1], there was an error regarding the FRAX® tool, which should be clarified as follows.

The article notes: “In fact, the WHO fracture risk predictor model (FRAX) based on data derived from nine cohorts from Europe, North America, Asia, and Australia includes low BMI as an important clinical predictor to predict 10-year probability of fracture [11].” However, the World Health Organization (WHO) did not develop, test, or endorse the FRAX tool or its recommendations [2]. The metabolic bone disease unit at the University of Sheffield that developed FRAX was a WHO Collaborating Centre from 1991 to 2010, but treatment guidelines must undergo a formal process before they can be endorsed by the WHO.
